# Associations between metabolic syndrome and type of dementia: analysis based on the National Health Insurance Service database of Gangwon province in South Korea

**DOI:** 10.1186/s13098-020-00620-5

**Published:** 2021-01-06

**Authors:** Yeo Jin Kim, Sang Mi Kim, Dae Hyun Jeong, Sang-Kyu Lee, Moo-Eob Ahn, Ohk-Hyun Ryu

**Affiliations:** 1 Department of Neurology, Hallym University-Chuncheon Sacred Heart Hospital, Hallym University College of Medicine, Chuncheon, Gangwon-do Republic of Korea; 2 Department of Big Data Analytics, Ewha Woman’s University, Seoul, Republic of Korea; 3 Research Institute for Gangwon, Chuncheon, Gangwon-do Republic of Korea; 4 Department of Psychiatry, Hallym University-Chuncheon Sacred Heart Hospital, Hallym University College of Medicine, Chuncheon, Gangwon-do Republic of Korea; 5 Department of Emergency Medicine, Hallym University-Chuncheon Sacred Heart Hospital, Hallym University College of Medicine, Chuncheon, Gangwon-do Republic of Korea; 6 Division of Endocrinology and Metabolism, Department of Internal Medicine, Hallym University-Chuncheon Sacred Heart Hospital, Hallym University College of Medicine, 77 Sakju-ro, Chuncheon, Gangwon-do 24253 Republic of Korea

**Keywords:** Metabolic syndrome, National Health Insurance Service, Dementia risk, Alzheimer’s disease, Vascular dementia

## Abstract

**Background:**

Metabolic syndrome is a cluster of conditions that occur together, increasing the risk of cardiovascular disease. However, the relationship between metabolic syndrome and dementia has remained controversial. Using nationwide population cohort data, we investigated the association between metabolic syndrome and dementia, according to the dementia type.

**Methods:**

We analyzed data of 84,144 individuals, in the aged group of more than 60 years, between January 1, 2009, to December 31, 2009, at Gangwon province by using the information of the (Korean) National Health Insurance Service. After eight years of gap, in 2017, we investigated the relationship between metabolic syndrome and dementia. We classified Dementia either as dementia of the Alzheimer type (AD) or vascular dementia (VD). AD and VD were defined as per the criteria of International Classification of Disease, Tenth Revision, Clinical Modification codes. Multiple logistic regression analyses examined the associations between metabolic syndrome or five metabolic syndrome components and dementia. Analyses included factors like age, sex, smoking, alcohol, physical inactivity, previous stroke, and previous cardiac disease.

**Results:**

Metabolic syndrome was associated with AD (OR = 11.48, 95% CI 9.03–14.59), not with VD. Each of five components of metabolic syndrome were also associated with AD. (high serum triglycerides: OR = 1.87, 95% CI 1.60–2.19; high blood pressure: OR = 1.85, 95% CI 1.55–2.21; high glucose: OR = 1.77, 95% CI 1.52–2.06; abdominal obesity: OR = 1.88, 95% CI 1.57–2.25; low serum high-density lipoprotein cholesterol: OR = 1.91, 95% CI 1.63–2.24) However, among components of metabolic syndrome, only the high glucose level was associated with VD. (OR = 1.26, 95% CI 1.01–1.56) body mass index (BMI), fasting glucose, and smoking were also associated with AD. (BMI: OR = 0.951, 95% CI 0.927–0.975; fasting glucose: OR = 1.003, 95% CI 1.001–1.005; smoking: OR = 1.020, 95% CI 1.003–1.039) A history of the previous stroke was associated with both AD and VD. (AD: OR = 1.827, 95% CI 1.263–2.644; VD: OR 2.775, 95% CI 1.747–4.406)

**Conclusions:**

Metabolic syndrome was associated with AD but not with VD. Patients with metabolic syndrome had an 11.48 times more likeliness to develop AD compared to those without metabolic syndrome. VD was associated only with several risk factors that could affect the vascular state rather than a metabolic syndrome. We suggested that the associations between metabolic syndrome and dementia would vary depending on the type of dementia.

## Background

Metabolic syndrome is a cluster of components that indicate overnutrition and includes five components that are high blood pressure (BP), high blood glucose, high serum triglycerides (TG), low serum high-density lipoprotein cholesterol (HDL-C), and abdominal obesity. Metabolic syndrome components like high blood pressure, blood glucose, abdominal obesity, and dyslipidemia are well-known factors associated with the occurrence of dementia in late life [1–4]. However, previous studies between metabolic syndrome and cognitive impairment were inconsistent. Some studies reported that metabolic syndrome was related to increased risk of cognitive impairment [5], while other studies reported no association between the two [6–8], and even some reported that metabolic syndrome decelerated cognitive impairment [9, 10].

Of the numerous dementia etiologies, dementia of the Alzheimer type is the most common type of dementia, about 60% of the cases [11]. Vascular dementia (VD) is the second most common type, accounting for about 20% of dementia [12]. Cardiovascular risk factors could affect the development of both types, but the extent of their influences would depend on the type of dementia. VD had a leading cause in most cases, such as ischemia, hemorrhage, anoxia, or hypoxia, while the causes of Alzheimer’s disease (AD) were not well understood [12]. Yet, previous studies that investigated the relationship between dementia and metabolic syndrome usually did not consider the type of dementia.

Gangwon province is located in the northeastern part of South Korea and is divided into two areas by the Mountains. It has an aging society with a relatively low population density in Korea. Although a large part of the population resides in the urban area, 11 out of 18 administrative areas have inadequate medical facilities because of the presence of mountains. That means this area is one of the most vulnerable areas of medical service in South Korea. The risk factors related to metabolic syndrome (cardiovascular and related diseases) are not well managed in this region; therefore, the disease’s characteristics might differ from other regions.

We hypothesized that metabolic syndrome would be more associated with the risk of dementia than its individual components due to the synergistic effect of vascular risk factors. We also thought that the risk of dementia associated with metabolic syndrome differed between AD and VD. Therefore, we analyzed the association of metabolic syndrome and its five components with the incidence of dementia (a gap of eight years), according to the dementia type, in a population-based sample in Gangwon province, South Korea.

## Methods

### Data source and study population

The present study was conducted using data from the South Korean National Health Insurance Service-National Sample Cohort (NHIS-NSC), which includes demographic information, medical use, disease information, lifestyle habits, and basic laboratory data [13, 14]. The NHIS registration is mandatory for all Koreans, and the NHIS database represents health information for almost all populations in Korea [15].

**Fig. 1:**
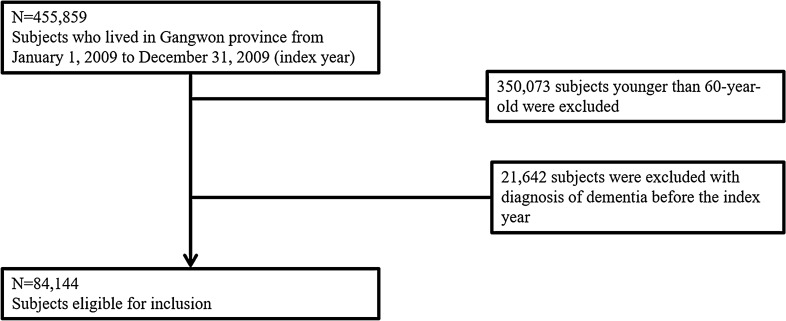
Flow chart of the study population

Patient data of Gangwon province in South Korea from January 1, 2009, to December 31, 2009, was included (n = 455,859). Of these patients, we selected only those aged more than 60 years (n = 105,786). Patients with dementia who was diagnosed before the index day were excluded (n = 21,642). In the end, 84,144 individuals were included in this study. (Fig. 1) We defined diagnoses using the International Classification of Disease, Tenth Revision, Clinical Modification (ICD-10-CM) codes.

### Standard protocol approvals, registrations, and patient consents

This study was approved by the Institutional Review Board of Chuncheon Sacred Heart Hospital, and all methods were performed in accordance with the approved guidelines and regulations.

### Definition of dementia

For defining dementia of the Alzheimer type (AD), the code of F00 or G30 was included, but F01, F02, F03, F051, and G31 were excluded. For the definition of vascular dementia (VD), codes of F02 were included. For defining dementia of any type of dementia, the code of F00, F01, F02, F03, G30, and G31 were included.

### Definition of metabolic syndrome

Individuals who met three or more of the five components were defined as having metabolic syndrome. Five components were abdominal obesity, high TG level, reduced HDL-C level, elevated BP, and elevated blood glucose [16]. Individuals having abdominal obesity were defined if waist circumference were over 90 cm in males and 80 cm in the female. Individuals having high TG level were defined if the serum TG level was over 150 mg/dL. Individuals having low HDL-C level were defined if serum HDL-C level was lower than 40 mg/dL in male and 50 mg/dL in the female. Individuals were defined as having elevated blood pressure if anti-hypertensive medications were prescribed or systolic blood pressure more than 130 mmHg and/or diastolic blood pressure more than 85 mmHg was recorded. Individuals having high blood glucose were defined if anti-diabetic drugs (insulins, sulfonylureas, metformin, meglitinides, thiazolidinediones, dipeptidyl peptidase-4 inhibitors, and α-glucosidase inhibitors) were prescribed or fasting serum glucose level was over 100 mg/dL.

### Statistical analysis

The baseline characteristics based on the data from the NHIS database were presented as mean values ± standard deviation (SD) for continuous variables and percentages for categorical variables. Differences between the metabolic syndrome group and no metabolic syndrome group were confirmed using the Student t-test for continuous variables and chi-square tests for categorical variables. The relationship of metabolic syndrome and each component of metabolic syndrome for dementia was evaluated using multiple logistic regression analysis. We ran three regression models. In model 1, we performed multiple logistic regression analyses with metabolic syndrome or each of the components as determinant and AD or VD as outcome variables after controlling for age and sex. In model 2, we performed multiple logistic regression analysis with age, sex, smoking, alcohol, physical inactivity, and metabolic syndrome. We also performed multiple logistic regression analysis with age, sex, smoking, alcohol, physical inactivity, and five metabolic syndrome components (high TG, high BP, high blood glucose, abdominal obesity, and low HDL-C). In model 3, we performed multiple logistic regression analysis with age, sex, smoking, alcohol, physical inactivity, previous stroke, previous cardiac disease, and metabolic syndrome or five metabolic syndrome components (high TG, high BP, high glucose, abdominal obesity, and low HDL-C). Again, the relationship of other vascular risk factors for dementia was evaluated using multiple logistic regression analysis. We also ran three regression models in this analysis. In model 1, we performed multiple logistic regression analyses with vascular risk factors as determinants and AD or VD as outcome variables after controlling for age and sex. In model 2, we performed multiple logistic regression analysis with age, sex, metabolic syndrome, BMI, systolic BP, diastolic BP, fasting glucose, and total cholesterol. In model 3, we performed multiple logistic regression analysis with age, sex, metabolic syndrome, BMI, systolic BP, diastolic BP, fasting glucose, total cholesterol, smoking, alcohol, physical inactivity, previous stroke, and previous cardiac disease. Disease risks were expressed as the odds ratio (OR) with a 95% confidence interval (95% CI). We defined statistical significance as p < 0.05. Statistical analyses were conducted with SPSS version 25 software (SPSS Inc., Chicago, IL, USA).

## Results

### Demographics and baseline characteristics

Detailed demographic and clinical characteristics of the participants were presented in Table 1. 40.2% of participants had metabolic syndrome. The mean age of the metabolic syndrome group was higher than the no metabolic syndrome group. The metabolic syndrome group had a higher proportion of females than the no metabolic syndrome group. The no metabolic syndrome group had a higher level of physical inactivity; however, they were also engaged in smoking and alcohol intake. The mortality rate of both groups was similar.

**Table 1:** Demographics and baseline characteristics

	Metabolic syndrome (n = 33,828)	No metabolic syndrome (n = 50,316)	p value
Mean age	67.42 ± 5.28	66.79 ± 5.11	< 0.0001
Sex, female number (%)^a^	23,020 (68.1)	25,088 (49.9)	< 0.0001
BMI	25.88 ± 2.96	23.68 ± 2.90	< 0.0001
Systolic BP	136.61 ± 15.15	126.54 ± 15.91	< 0.0001
Diastolic BP	82.03 ± 10.12	77.18 ± 9.97	< 0.0001
Fasting glucose	112.14 ± 31.27	96.70 ± 20.45	0.001
Total cholesterol	200.80 ± 46.30	194.55 ± 38.46	< 0.0001
Smoking, pack-year	1.33 ± 4.89	2.16 ± 5.99	< 0.0001
Alcohol, cup/week	1.20 ± 2.94	1.54 ± 3.20	< 0.0001
Previous stroke (%)^a^	804 (2.38)	876 (1.74)	< 0.0001
Previous cardiac disease (%)^a^	2137 (62.2)	2513 (5.8)	< 0.0001
Previous HTN (%)^a^	18,062 (62.2)	17,180 (39.6)	< 0.0001
Previous DM (%)^a^	6969 (24.1)	4102 (9.5)	< 0.0001
Previous dyslipidemia (%)^a^	2225 (7.7)	2493 (5.8)	< 0.0001
Physical inactivity (%)^a^	5487 (29.8)	9483 (35.0)	< 0.0001
High TG (%)^a^	21,746 (64.3)	7617 (15.1)	< 0.0001
High BP (%)^a^	27,571 (81.5)	22,848 (45.4)	< 0.0001
High glucose (%)^a^	22,422 (33.7)	12,804 (74.6)	< 0.0001
Abdominal obesity (%)^a^	26,806 (79.2)	14,939 (29.7)	< 0.0001
Low HDL-C (%)^a^	19,768 (58.4)	8,377 (16.65)	< 0.0001

Data are mean ± SD unless otherwise indicated

*BMI* body mass index, *SD* standard deviation, *HTN* hypertension, *DM* diabetes mellitus, *TG* triglyceride, *BP* blood pressure, *HDL-C* high-density lipoprotein cholesterol

^a^ Number (%)

After eight years, of the 33,828 patients with metabolic syndrome, 1380 patients (4.08%) converted to AD, and 335 patients (0.99%) converted to VD. Of the 50,316 patients without metabolic syndrome, 176 patients (0.35%) converted to AD, and 380 patients (0.76%) converted to VD.

### Association between metabolic syndrome and AD

Table 2 showed the risk of AD according to the metabolic syndrome and component of the definition of metabolic syndrome.

**Table 2:** Metabolic syndrome and odds of dementia in Alzheimer’s type

	Model 1		Model 2		Model 3	
	OR (95% CI)	p value	OR (95% CI)	p value	OR (95% CI)	p value
MetS	10.58 (9.02, 12.41)	< 0.0001	12.03 (9.58, 15.10)	< 0.0001	11.48 (9.03, 14.59)	< 0.0001
Hight TG	2.64 (2.38, 2.92)	< 0.0001	2.00 (1.73, 2.31)	< 0.0001	1.87 (1.60, 2.19)	< 0.0001
High BP	1.87 (1.67, 2.11)	< 0.0001	1.78 (1.51, 2.10)	< 0.0001	1.85 (1.55, 2.21)	< 0.0001
High glucose	2.31 (2.08, 2.54)	< 0.0001	1.84 (1.60, 2.12)	< 0.0001	1.77 (1.52, 2.06)	< 0.0001
Abdominal obesity	2.37 (2.10, 2.67)	< 0.0001	1.77 (1.50, 2.09)	< 0.0001	1.88 (1.57, 2.25)	< 0.0001
Low HDL-C	2.33 (2.10, 2.59)	< 0.0001	1.95 (1.69, 2.26)	< 0.0001	1.91 (1.63, 2.24)	< 0.0001

Model 1: separate models associated each exposure variable with AD with adjustment for age and sex

Model 2: single model including age, sex, smoking, alcohol, physical inactivity, and metabolic syndrome or 5 metabolic syndrome components (high TG, high BP, high glucose, abdominal obesity, low HDL-C)

Model 3: single model including age, sex, smoking, alcohol, physical inactivity, previous stroke, previous cardiac disease, and metabolic syndrome or 5 metabolic syndrome components (high TG, high BP, high glucose, abdominal obesity, low HDL-C)

*OR* odds ratio, *CI* confidence interval, *MetS* metabolic syndrome, *TG* triglyceride, *BP* blood pressure, *HDL-C* high-density lipoprotein cholesterol

Metabolic syndrome was associated with AD. (OR 11.48, 95% CI 9.03, 14.59, p < 0.0001). Each of the five components of metabolic syndrome were also associated with AD. Patients with high TG had 1.87-fold odds of AD (OR 1.87, 95% CI 1.60, 2.19, p < 0.001). Patients with high BP had 1.85-fold odds of AD (OR 1.85, 95% CI 1.55, 2.21, p < 0.0001). Patients with high glucose levels had 1.77-fold odds of AD (OR 1.77, 95% CI 1.52, 2.06, p < 0.0001). Patients with abdominal obesity had 1.88-fold odds of AD (OR 1.88, 95% CI 1.57, 2.25, p < 0.0001). Patients with low HDL-C had 1.91-fold odds of AD (OR 1.91, 95% CI 1.63, 2.24, p < 0.0001).

### Association between metabolic syndrome and VD

Table 3 showed the risk of VD according to the metabolic syndrome and component of the definition of metabolic syndrome.

**Table 3:** Metabolic syndrome and odds of vascular dementia

	Model 1		Model 2		Model 3	
	OR (95% CI)	p value	OR (95% CI)	p value	OR (95% CI)	p value
MetS	1.08 (1.02, 1.15)	0.006	1.17 (0.95, 1.45)	0.144	1.17 (0.94, 1.47)	0.158
Hight TG	1.03 (0.88, 1.20)	0.709	0.95 (0.76, 1.19)	0.631	0.90 (0.71, 1.14)	0.392
High BP	1.07 (0.92, 1.25)	0.404	1.31 (1.05, 1.64)	0.019	1.25 (0.99, 1.58)	0.066
High glucose	1.30 (1.11, 1.50)	0.001	1.29 (1.04, 1.59)	0.019	1.26 (1.01, 1.56)	0.042
Abdominal obesity	1.08 (0.92, 1.21)	0.362	0.97 (0.77, 1.21)	0.754	0.99 (0.78, 1.26)	0.939
Low HDL-C	1.03 (0.88, 1.20)	0.736	0.97 (0.77, 1.22)	0.806	0.97 (0.76, 1.23)	0.785

Model 1: separate models associated each exposure variable with VD with adjustment for age and sex

Model 2: single model including age, sex, smoking, alcohol, physical inactivity, and metabolic syndrome or 5 metabolic syndrome components (high TG, high BP, high glucose, abdominal obesity, low HDL-C)

Model 3: single model including age, sex, smoking, alcohol, physical inactivity, previous stroke, previous cardiac disease, and metabolic syndrome or 5 metabolic syndrome components (high TG, high BP, high glucose, abdominal obesity, low HDL-C)

*OR* odds ratio, *CI* confidence interval, *MetS* metabolic syndrome, *TG* triglyceride, *BP* blood pressure, *HDL-C* high-density lipoprotein cholesterol

Metabolic syndrome was not associated with VD (OR 1.17, 95% CI 0.94, 1.47, p = 0.158). Among the five components of metabolic syndrome, high glucose was associated with VD. Patients with high glucose had 1.26-fold odds of VD (OR 1.26, 95% CI 1.01, 1.56, p = 0.042). High TG, high BP, abdominal obesity, and low HDL-C were not associated with VD.

### Association between metabolic syndrome and Any type of dementia

Table 4 showed the risk of any type of dementia according to the metabolic syndrome and component of the definition of metabolic syndrome.

**Table 4:** Metabolic syndrome and odds of any type of dementia

	Model 1		Model 2		Model 3	
	OR (95% CI)	p value	OR (95% CI)	p value	OR (95% CI)	p value
MetS	1.44 (1.37, 1.50)	< 0.0001	1.47 (1.39, 1.57)	< 0.0001	1.47 (1.37, 1.57)	< 0.0001
Hight TG	1.25 (1.20, 1.31)	< 0.0001	1.19 (1.12, 1.27)	< 0.0001	1.16 (1.08, 1.24)	< 0.0001
High BP	1.04 (1.00, 1.09)	0.073	1.04 (0.97, 1.10)	0.264	1.06 (0.99, 1.14)	0.092
High glucose	1.19 (1.14, 1.24)	< 0.0001	1.12 (1.06, 1.20)	< 0.0001	1.13 (1.05, 1.20)	< 0.0001
Abdominal obesity	1.22 (1.16, 1.28)	< 0.0001	1.17 (1.09, 1.24)	< 0.0001	1.17 (1.09, 1.26)	< 0.0001
Low HDL-C	1.18 (1.12, 1.23)	< 0.0001	1.12 (1.05, 1.19)	0.001	1.10 (1.03, 1.18)	0.007

Model 1: separate models associated each exposure variable with any type of dementia with adjustment for age and sex

Model 2: single model including age, sex, smoking, alcohol, physical inactivity, and metabolic syndrome or 5 metabolic syndrome components (high TG, high BP, high glucose, abdominal obesity, low HDL-C)

Model 3: single model including age, sex, smoking, alcohol, physical inactivity, previous stroke, previous cardiac disease, and metabolic syndrome or 5 metabolic syndrome components (high TG, high BP, high glucose, abdominal obesity, low HDL-C)

*OR* odds ratio, *CI* confidence interval, *MetS* metabolic syndrome, *TG* triglyceride, *BP* blood pressure, *HDL-C* high-density lipoprotein cholesterol

Metabolic syndrome was associated with any type of dementia (OR 1.47, 95% CI 1.37, 1.57, p < 0.0001). Among the five components of metabolic syndrome, high TG, high glucose, abdominal obesity, and low HDL-C were associated with any type of dementia. Patients with high TG had 1.16-fold odds of any type of dementia (OR 1.16, 95% CI 1.08, 1.24, p < 0.0001). Patients with high glucose had 1.13-fold odds of any type of dementia (OR 1.13, 95% CI 1.05, 1.20, p < 0.0001). Patients with abdominal obesity had 1.17-fold odds of any type of dementia (OR 1.17, 95% CI 1.09, 1.26, 0 < 0.0001). Patients with low HDL-C had 1.10-fold odds of any type of dementia (OR 1.10, 95% CI 1.03, 1.18, p = 0.007).

### Association between other cardiovascular risk factors included continuous metabolic parameters and dementia

BMI, fasting glucose, smoking, and previous stroke history were found associated with AD (Table 5). Patients with each 1-unit kg/m^2^ higher BMI had 0.951-fold odds of AD. (OR 0.951, 95% CI 0.927, 0.975, p < 0.0001) Patients with each 1-unit mg/dL higher fasting glucose had 1.003-fold odds of AD. (OR 1.003, 95% CI 1.001, 1.005, p = 0.003) Patients with each 1-year higher pack/year smoking had 1.020-fold odds of AD (OR 1.020, 95% CI 1.003, 1.039, p = 0.024). Patients with having previous stroke history had 1.827-fold odds of AD. (OR 1.827, 95% CI 1.263, 2.644, p = 0.001).

**Table 5:** Odds of the dementia in Alzheimer’s type or VD according to vascular risk factors

	Alzheimer’s disease						Vascular dementia					
	Model 1		Model 2		Model 3		Model 1		Model 2		Model 3	
	OR (95% CI)	p value	OR (95% CI)	p value	OR (95% CI)	p value	OR (95% CI)	p value	OR (95% CI)	p value	OR (95% CI)	p value
BMI	1.059 (1.042, 1.075)	< 0.0001	0.958 (0.941, 0.975)	< 0.0001	0.951 (0.927, 0.975)	< 0.0001	1.008 (0.984, 1.032)	0.528	0.998 (0.973, 1.023)	0.857	0.988 (0.952, 1.024)	0.504
Systolic BP	1.014 (1.011, 1.017)	< 0.0001	1.000 (0.995, 1.004)	0.882	0.999 (0.992, 1.005)	0.651	1.000 (0.996, 1.005)	0.855	0.994 (0.988, 1.000)	0.063	0.999 (0.990, 1.008)	0.788
Diastolic BP	1.014 (1.009, 1.018)	< 0.0001	0.996 (0.990, 1.002)	0.214	1.001 (0.991, 1.010)	0.916	1.007 (0.999, 1.014)	0.071	1.002 (1.002, 1.022)	0.019	1.004 (0.990, 1.019)	0.575
Fasting glucose	1.009 (1.008, 1.011)	< 0.0001	1.004 (1.002, 1.005)	< 0.0001	1.003 (1.001, 1.005)	0.003	1.005 (1.003, 1.007)	< 0.0001	1.002 (1.002, 1.007)	< 0.0001	1.004 (1.001, 1.008)	0.012
Total cholesterol	1.001 (1.000, 1.002)	0.003	1.001 (1.000, 1.002)	0.211	1.000 (0.998, 1.002)	0.793	1.000 (0.999, 1.002)	0.662	0.999 (0.999, 1.002)	0.793	1.000 (0.997, 1.003)	0.998
Smoking	1.007 (0.994, 1.021)	0.282			1.020 (1.003, 1.039)	0.024	0.928 (0.892, 0.966)	< 0.0001			0.988 (0.962, 1.014)	0.352
Alcohol	0.995 (0.969, 1.021)	0.694			0.979 (0.940, 1.019)	0.302	0.972 (0.936, 1.011)	0.155			1.014 (0.972, 1.056)	0.525
Physical inactivity	0.935 (0.945, 1.163)	0.415			0.997 (0.838, 1.188)	0.977	0.974 (0.836, 1.136)	0.741			0.898 (0.700, 1.153)	0.399
Previous stroke	2.248 (1.757, 2.874)	< 0.0001			1.827 (1.263, 2.644)	0.001	2.461 (1.753, 3.445)	< 0.0001			2.775 (1.747, 4.406)	< 0.0001
Previous cardiac disease	1.213 (1.004, 1.467)	0.046			1.088 (0.836, 1.416)	0.532	0.825 (0.598, 1.139)	0.242			0.702 (0.434, 1.136)	0.150

Model 1: separate models associated each exposure variable with AD or VD with adjustment for age and sex

Model 2: single model including age, sex, metabolic syndrome, BMI, systolic BP, diastolic BP, fasting glucose, total cholesterol

Model 3: single model including age, sex, metabolic syndrome, BMI, systolic BP, diastolic BP, fasting glucose, total cholesterol, smoking, alcohol, physical inactivity, previous stroke and previous cardiac disease

*OR* odds ratio, *CI* confidence interval, *BMI* body mass index, *BP* blood pressure

Fasting glucose and previous stroke history were associated with VD. Patients with each 1-unit mg/dL higher fasting glucose had 1.004-fold odds of VD (OR 1.004, 95% CI 1.001, 1.008, p = 0.012). Patients with having previous stroke history had 2.775-fold odds of VD. (OR 2.775, 95% CI 1.747, 4.406, p < 0.0001).

## Discussion

We investigated how metabolic syndrome and its components were associated with the development of dementia after eight years. Metabolic syndrome was associated with AD, while it was not associated with VD. Rather than that, high fasting glucose and previous stroke history were associated with VD. BMI, fasting glucose, smoking, and previous stroke were also associated with AD.

The odds of metabolic syndrome were much greater than the sum of the odds of five components in AD. This might mean that the association between metabolic syndrome and AD was greater than the sum of the association between each component and AD. Metabolic syndrome represented a chronic state of inflammation, hyperinsulinemia, dyslipidemia, dysglycemia, vascular injury, and oxidative stress linked to AD [17]. Amyloid beta (Aβ) deposition initiated an immune response intended to clear the amyloid plaque [18]. However, this response also stimulated the cytokine cascade and reactive oxygen species (ROS), leading to neurodegeneration [19]. Furthermore, the inflammatory cascade altered phosphorylation of tau protein along with the oxidative injury to the neurons. The immune response induced by amyloid, in addition to the chronic inflammatory state due to metabolic syndrome, exacerbated the AD pathogenic process, leading to the progression of AD. [20, 21] Metabolic syndrome also induced oxidative stress and increased ROS production. [22] The circulating lipid and glucose imbalance combined with ROS enhanced lipoperoxidation led to the dysfunction of the antioxidant system. This usually caused vascular injury and blood–brain barrier (BBB) dysfunction, affecting amyloid and tau accumulation and chronic hypoperfusion, leading to neuronal damage [17].

Previous studies that investigated the association between metabolic syndrome and dementia reported inconsistent results. Some studies showed an association between metabolic syndrome and AD [23, 24], while other studies showed no association [7, 25–27]. French Three-City cohort and Italian Longitudinal Study on the aging study showed that metabolic syndrome increased VD risk, not AD [26, 27]. Other studies showed no association between metabolic syndrome and dementia [7, 9, 28]. However, the associations between metabolic syndrome and cognitive impairment varied with the study population characteristics, various criteria used to define metabolic syndrome, diverse approaches used to assess cognitive function, and different follow-up periods [6, 24]. Also, meta-analyses showed inconsistent associations between metabolic syndrome and dementia. It was reported that there was no association between metabolic syndrome and incident dementia or AD. Metabolic syndrome only increased the risk of pure VD [8]. Another meta-analysis showed that metabolic syndrome increased the risk of conversion from mild cognitive impairment to all-cause dementia [29]. Another study reported that metabolic syndrome decreased the risk of AD [10]. The association between metabolic syndrome and dementia could vary depending on age, follow-up period, and genetic susceptibility [30]. Especially, Apolipoprotein E4 (APOE4), the strongest genetic risk factor for late onset AD, was also associated with metabolic syndrome [31]. Apolipoprotein was a lipid carrying lipoprotein, which interacted with amyloid beta [32]. Influence of APOE4 on the cerebral cortex varied with age [33], and this might be related to inconsistent previous research results on the relationship between metabolic syndrome and risk of dementia.

In this study, all the five components of metabolic syndrome were associated with AD, while metabolic syndrome components except high blood glucose were not associated with VD. Although mid-life hypertension [34], mid-life dyslipidemia [35, 36], and mid-life obesity [37] were consistently related to late-life dementia, previous studies of the association between late-life vascular risk factors and dementia reported mixed results [34, 37–39]. Meanwhile, ischemia could cause up-regulation of amyloid precursor protein expression in human brains [40]. The co-existence of cerebrovascular pathology and amyloid pathology could increase clinically incident dementia [41]. Even though we removed the patients who had AD and VD code in AD diagnosis, it might be possible to have co-pathology in AD. AD patients who had vascular pathology could develop clinical symptoms easier than VD patients having only vascular pathology.

In this study, patients with metabolic syndrome had an 11.48 times more likeliness to develop AD compared to those without metabolic syndrome, and there was no association between metabolic syndrome and VD. In a previous study, metabolic syndrome was associated with accelerated amyloid beta accumulation in the elderly with amyloid deposition [42]. Therefore, the combination of amyloid deposition and vascular risk factors might have increased AD incidence in individuals with metabolic syndrome. Even taking the combination of amyloid deposition and vascular risk factor into account, this study showed a very large OR value than other studies, which might be due to racial differences. Most of the previous studies have been conducted either on the European or American populations [8]. Since this study was only on the South Korean population, one of the Asian races, there was a possibility that there might be differences in the associations between metabolic syndrome and dementia according to races.

Among components of metabolic syndrome, only high glucose level was associated with both AD and VD, while others showed association only with AD. Patients with 10 mg/dL higher fasting glucose had a 0.3% increased risk of AD and a 0.4% increased risk of VD. This is consistent with previous studies showing the association between diabetes and increased risk of dementia, including all-cause dementia, AD and VD [43]. In addition, a previous study reported that, even in patients without diabetes, higher average glucose levels were associated with an increased risk of dementia [44]. Previous studies reported that diabetes mellitus not only affected cognitive decline [45] but was also associated with a high risk of cerebrovascular disease, including high white matter hyperintensities volume [46] and cerebral infarcts [47, 48]. Impaired insulin signaling and glucose metabolism in the brain were factors that were related to AD pathogenesis. Insulin modulated Aβ protein precursor expression and processing [49]. Insulin not only regulated glucose and lipid metabolism in the brain but also regulated neural development and neuronal activities associated with learning and memory [50]. Insulin receptors were expressed in the brain, particularly in memory registration-related areas, such as the cerebral cortex, hippocampus, hypothalamus, and amygdala [51]. Therefore, impairment of insulin signaling led to the pathologic processes of AD. Besides, we assumed that glucose metabolism would be more closely related to the mechanism that caused cognitive impairment after the vascular event than other components. Although previous studies showed that other components of metabolic syndromes, such as hypertension [52], dyslipidemia [53], and abdominal obesity [54], also increase ischemic stroke, our study showed that these factors were not associated with VD. Another study also showed that high glucose was the most significant component associated with cognitive impairment [55].

The previous stroke was also a factor that affected both AD and VD. Patients having stroke history were 1.8 times more likely to develop AD and 2.8 times more likely to develop VD. The association between stroke and dementia was already reported in several studies. Ischemic stroke was a risk factor for developing AD and VD [56, 57]. Stroke doubled the risk of dementia, and approximately 20% of stroke patients went on to develop cognitive dysfunction within 3 years [58]. Ischemic stroke led to pathophysiological processes that contributed to ischemic cell damage [59]. Stimulation of the inflammatory process, free radical production, excitotoxicity, disruption of sodium and calcium influx, enzymatic changes, endothelin release, delayed coagulation, activation of platelets and leukocytes, and endothelial dysfunction were the pathophysiological reactions resulting from the onset of stroke [60]. Several studies reported a synergistic relationship between ischemic stroke and AD. Postmortem studies showed that individuals with AD pathology with cerebral infarction had a markedly increased risk of dementia than those with AD pathology without infarcts [61, 62]. Stroke was suggested as a contributing factor to AD pathological changes, including selective brain atrophy and accumulation of abnormal protein such as Aβ [63]. A previous study also provided evidence that stroke led to cognitive dysfunction more rapidly in patients with AD [64]. Besides, VD was the severest form of vascular cognitive impairment [12], and it resulted from subclinical vascular brain injury and stroke. Major stroke and minor stroke, and even transient ischemic attack were known to increase the risk of dementia [65].

As per our study, while patients with abdominal obesity had a higher risk of AD, patients with higher BMI were less prone to AD. This discrepancy was probably due to BMI not being a perfect indicator of obesity. BMI could not distinguish between fat and lean body mass, and because lean body mass decreased due to aging, the increase in BMI in the elderly might be due to an increase in lean body mass rather than an increase in fat [66]. On the other hand, the waist circumference used as the criterion for abdominal obesity in this study was a more accurate indicator of abdominal visceral fat level, especially in old age. [67] While adipose tissue secreted leptin, which had a good effect on brain function, [68] abdominal visceral fat increased the risk of dementia through chronic inflammation and neuronal degeneration. [69–71] Through this study, it could be assumed that abdominal visceral fat level was a more specific risk factor that increased the risk of dementia.

AD was also found associated with smoking. Patients with 10-year higher pack/year smoking had a 20% increased risk of AD. It was controversial whether smoking has any harmful effect on degenerative diseases. Previously some studies reported that smoking had a protective effect on degenerative diseases [72–74]. Researches even showed that nicotine had a neuroprotective and anti-aging effect [75, 76]. However, nowadays, smoking was attributed as a risk factor for AD [77]. Smoking increased oxidative stress and might have indirect effects on several vascular, inflammatory, and degenerative processes [78, 79]. If smoking particles were inhaled, they stimulated ROS production and entered the brain via blood. Smoking-related cerebral oxidative stress was a potential mechanism to accelerate AD pathology and increasing the risk for AD [80]. Smoking also impaired nitric oxide synthesis in cerebral vascular endothelial cells leading to interference with cerebral blood flow and glucose metabolism in the brain. [81, 82] It induced cerebral hypoperfusion and promoted the synthesis of Aβ [83]. Smoking stimulated the release of proinflammatory cytokines and immune system-mediated products, causing an increase in Aβ accumulation and tau phosphorylation, hallmarks of AD pathology [84].

Our study had some limitations. First, our study used claimed data, according to which we classified the types of dementia, but the actual amyloid burden was unknown. However, in order to distinguish the pure dementia type, those with codes of both AD and VD were excluded. Second, since we only investigated the presence of dementia eight years later, there was no consideration of what happened during the eight years and at what point dementia occurred during the eight years. Besides, there was no consideration for efforts to overcome metabolic syndrome. Some might have tried to treat metabolic syndrome, others might not, but the effect of improvement of metabolic syndrome was not known in our study. Third, there was no consideration for the duration of metabolic syndrome. The association with dementia might vary depending on how long metabolic syndrome components have existed, which were not known in our study. Therefore, further study considering the duration of metabolic syndrome is required. Finally, as the cohort only included the specific region of Korea, further investigation in other regions or population is required to generalize these findings.

Despite these limitations, we investigated the association between metabolic syndrome and different types of dementia and the association between components of metabolic syndrome and dementia, using population-based data. Therefore, we hope to provide new clinical insight into the association between metabolic syndrome and type of dementia with implications for considering different pathophysiology.

## Conclusions

Metabolic syndrome was associated with AD, while it was not associated with VD. Patients with metabolic syndrome had an 11.48 times more likeliness to develop AD compared to those without metabolic syndrome. Therefore, the association between metabolic syndrome and dementia would be different depending on the type of dementia.

## Data Availability

The original anonymized data used in this analysis was obtained from the NHIS of South Korea. The dataset from NHIS is not publicly available due to restricted access. However, any researcher requiring access to the data can obtain it directly through a license agreement, including the payment of appropriate license fees.

## Abbreviations

AD: Dementia of the Alzheimer type
VD: Vascular dementia
BMI: Body mass index
BP: Blood pressure
TG: Triglycerides
HDL-C: High-density lipoprotein cholesterol
NHIS: (Korean) National Health Insurance Service
NHIS-NSC: National Health Insurance Service-National Sample Cohort
ICD-10-CM: International Classification of Disease, Tenth Revision, Clinical Modification
SD: Standard deviation
OR: Odds ratio
95% CI: 95% Confidence interval
Aβ: Amyloid beta
ROS: Reactive oxygen species
BBB: Blood–brain barrier
